# Factors associated with device-based measured physical activity and sedentary behavior in a cross-sectional citizen science study of adolescents during the COVID-19 pandemic

**DOI:** 10.1186/s12889-025-25776-5

**Published:** 2025-12-05

**Authors:** Katharina Nimptsch, Yang Jiao, Lethizia Oliver-Stanley, Jonas Widmann, Lina Jaeschke, Astrid Steinbrecher, Kieran Dowd, Tobias Pischon

**Affiliations:** 1 Max Delbrück Center for Molecular Medicine in the Helmholtz Association (MDC), Molecular Epidemiology Research Group, Berlin, Germany; 2 Julius-Maximilians-Universität Würzburg, Institute of Clinical Epidemiology and Biometry, Würzburg, Germany; 3 Department of Sport and Health Sciences, Technological University of the Shannon, Athlone, Ireland; 4 Max Delbrück Center for Molecular Medicine in the Helmholtz Association (MDC), Biobank Technology Platform, Berlin, Germany; 5 Charité-Universitätsmedizin Berlin, Corporate Member of Freie Universität Berlin and Humboldt-Universität zu Berlin, Berlin, Germany

**Keywords:** Citizen science, Accelerometers, Physical activity, Sedentary behavior, Adolescents, Influencing factors

## Abstract

**Background:**

Insufficient physical activity (PA) and extensive sedentary behavior (SB) in adolescents has been related to adverse outcomes related to physical fitness, body weight as well as social and psychological health indicators. Furthermore, these behaviors tend to track from adolescence to adulthood, thereby affecting adult chronic disease risk. The knowledge of factors associated with PA and SB is needed particularly in adolescents, in order to plan public health strategies aiming at increasing PA and reducing SB. The aim of this Citizen Science project (conducted between February 2020 and June 2021) was to work together with young citizens on a school-based epidemiologic study assessing device-based measured PA and SB in students (grades 8 or higher) to identify associated factors based on both established as well as co-created questionnaires.

**Methods:**

In 12 school classes from Berlin and Brandenburg, students were as Citizen Scientists involved in the co-creation of class-specific questionnaires by collecting factors that may influence their PA and SB. Students participating in the study wore thigh-worn accelerometers (activPAL) for seven consecutive days and subsequently completed established as well as the newly developed class-specific questionnaires to ascertain potential influencing factors of PA and SB. Multilevel linear regression models were used to identify factors associated with time spent in moderate-to-vigorous physical activity (MVPA) and SB.

**Results:**

Accelerometry data with at least four recorded days were available for 119 students (783 recorded days). In models adjusted for age, sex and parental socioeconomic status, high traffic safety around the school (14.8 min/day, 95% CI 0.9, 28.7) and higher degree school type (10.7 min/day, 95% CI 1.7; 19.8) were associated with more time spent in MVPA. From the class-specific questionnaires developed based on input from students, the feeling of being exhausted after school and homework was associated with less time spent in MVPA, while internal motivation to be physically active, active hobbies and working out regularly at a gym were associated with more time spent in MVPA. The potential influencing factors under investigation were not associated with sedentary time (except female sex, which was related to lower sedentary time).

**Conclusions:**

Although our findings warrant confirmation in larger samples, this Citizen Science study points to potential action points that may be targeted in public health interventions aimed at increasing PA to improve health in adolescents.

**Supplementary Information:**

The online version contains supplementary material available at 10.1186/s12889-025-25776-5.

## Introduction

Low levels of physical activity (PA) and excessive sedentary behavior (SB) are independent risk factors for cardiometabolic health [1, 2]. Adolescence (age range between childhood and adulthood, usually between 10 and 19 years [3]), is a time period characterized by biological growth, physiological changes as well as psychosocial transitions and may be a critical time window determining future health. PA and SB in adolescence may influence adult health by tracking of activity behavior from adolescence to adulthood, where it may influence adult health, while it also influences adult health through short-term benefits of adolescent physical activity on cardiovascular risk factors, such as blood pressure, which plays a role in development of chronic disease later in life [4, 5]. In addition, physical inactivity and sedentary behavior in adolescents have been related to poor social and psychological health indicators [6–8]. In order to prevent the adverse health and psychosocial affects associated with insufficient PA and excessive sedentary time, knowledge on influencing factors for PA and SB are needed, particularly in adolescents, in order to implement public health measures to increase PA and reduce SB in a beneficial way.

Based on self-reported data, it has been estimated that 81% of school-going adolescents globally (11–17 years) were insufficiently physically active [9], i.e. did not meet the World Health Organization (WHO) recommendation of at least 60 min/day of moderate to vigorous physical activity (MVPA) [10]. Observational studies have shown that different personal, home-related, and school-related factors are associated with PA and SB in adolescents. Boys were consistently shown to be more physically active than girls in several studies [11], and a lower compared to higher parental socio-economic status (SES) was associated with higher sedentary times [12]. For the school environment, access to PA programs and equipment has been positively associated with PA in adolescents in a number of studies [13], while permission to use media devices has been positively associated with sitting time during school [14].

However, established questionnaires ascertaining potential influencing factors of PA and SB may miss certain factors considered as important by adolescents themselves. Therefore, involving young people in the questionnaire development may improve knowledge gain in studying influencing factors of PA and SB in adolescents. Citizen Science is a growing field in open science that encompasses the active participation of the public in scientific processes, providing multiple opportunities to generate innovative data, develop new scientific questions, and incorporate knowledge and input from society into research to shape the way different topics are viewed [15]. This concept may also be applied in public health research [16]. Questionnaire co-creation within a Citizen Science approach is particularly appealing in public health research addressing adolescents, because involving adolescents may provide crucial information content-wise (i.e. which questionnaire-items to consider), but also language-wise, because language and conceptualization may differ substantially from adults [17]. Involving students as citizen scientists in questionnaire development has previously been shown as a valuable tool to identify relevant and understudied influencing factors of attention [18].

Therefore, the aim of this research project was to work together with young citizens on a school-based epidemiologic study assessing device-based measured PA and SB in students (grades 8 or higher) to identify associated factors based on both established as well as co-created questionnaires. We hypothesized that male sex, higher SES and an activity friendly (school) environment are associated with higher PA and/or lower SB. Regarding the questionnaire co-creation, we hypothesized that we would identify factors beyond established associated factors that may potentially also be related to PA and SB.

## Methods

### Study design and recruitment

SMOVE stands for “Science that makes me move” and was one of two Citizen Science projects embedded in the open science project ORION (Open Responsible research and Innovation to further Outstanding kNowledge), funded by the European Union’s Horizon 2020 research and innovation program. SMOVE is a cross-sectional school-based epidemiologic study conducted in Berlin and Brandenburg, Germany. As a Citizen Science project, students were actively involved in different phases of the scientific research in order to build a cooperation between science and society and to generate new knowledge together. The role of students as citizen scientists was (i) to develop together with scientists a questionnaire to assess potential influencing factors of PA and SB and (ii) participate in the data collection as well as the analysis and interpretation of the data.

First contact with the schools were established by a targeted mailing campaign to schools/teachers in Berlin and Brandenburg, followed by teacher’s information workshops and signature of a participation agreement form by school principals. Overall, seven schools in Berlin (10 school classes, grade 8 and higher) and two schools in Potsdam, Brandenburg (2 school classes, grade 8th and higher) returned the signed forms and participated in the SMOVE project between February 2020 and June 2021. Participating schools’ teachers received the informed consent forms for students and their parents, and also for teachers (required in Brandenburg only), and the dates for the school visits were agreed.

All participating students and their parents (for students < 18 years) provided informed consent/assent for their participation in the project. SMOVE was conducted as an anonymized study with only teachers being able to connect the study identification numbers (pseudonyms) with the participating students. The study was approved by the local school authorities as well as ethically approved by the Charité – Universitätsmedizin Berlin.

### Instruments

Questionnaires for students (PA and SB, factors potentially associated with PA and SB identified from literature reviews (e.g., age, sex, media use, transportation, environmental factors)), their parents (SES, own media use, and self-reported PA behaviors), and teachers (school-specific potential influencing factors of PA and SB) were developed to capture established factors potentially associated with PA and SB based on previous research [14, 19]. The questionnaires for students were supplemented by additional questions developed by students in class through interaction with the scientists (i.e., questions ascertaining to factors students deem valuable to the analysis of the generated data on PA and SB).

PA and SB were assessed via thigh-worn activPAL accelerometers (activPAL3 micro, PAL Technologies Ltd., Glasgow, United Kingdom). The activPAL was worn on the midline of the anterior aspect of the right thigh for seven consecutive days and was only removed when submerged in water (i.e., bathing, swimming). Due to the position on the thigh, the activPAL device is specifically sensitive to quantify sedentary time, distinguishing between sitting/lying and standing with a high level of accuracy compared to other (e.g. hip-worn) devices [20]. In order to supplement and optimize the data output of the activPAL device, a diary was provided to the students to record periods they did not wear the devices as well as to report sleeping/bed phases.

### Data collection

**Fig. 1:**
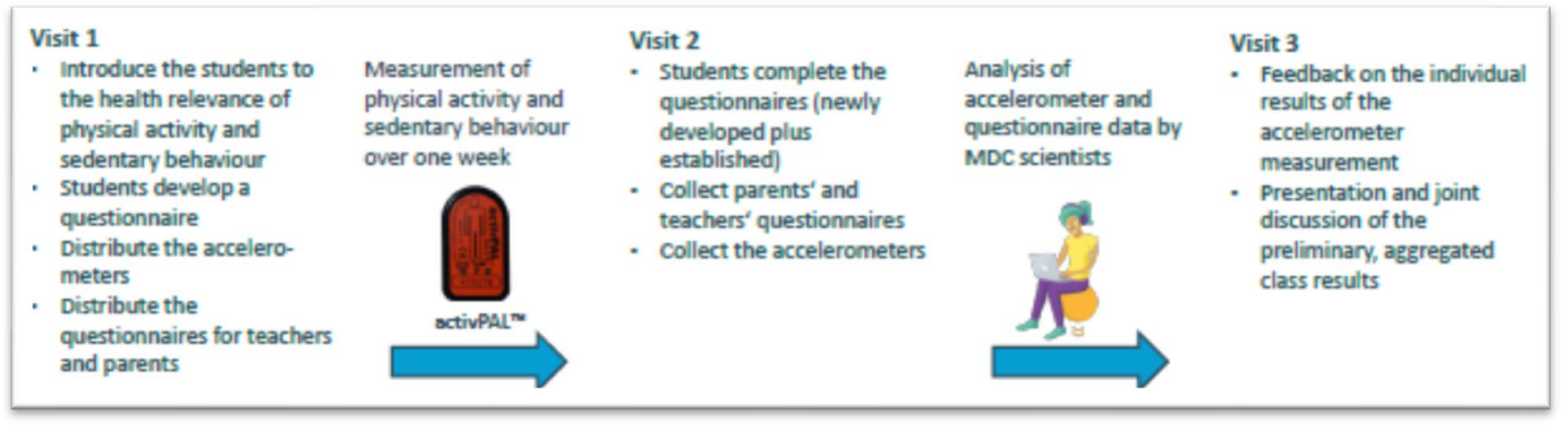
Practical field phase of SMOVE

The practical field phase of SMOVE consisted of up to three school visits (Fig. 1). During the first visit, the school class was introduced to the importance of PA and SB to health. The class was then divided into smaller groups to discuss factors that, in their opinion, influence their PA and SB. All potential influencing factors were then discussed by the whole class and ranked in order of importance, providing a basis to create new class-specific questionnaires. Further, during the first visit, the thigh-worn activPAL accelerometer devices were distributed. Questionnaires for teachers and parents were also distributed during the first school visit.

Between the first and second visit, the researchers converted the factors considered most important into individual questionnaires for each class. Only questions that were not already covered by the established core student questionnaire were included. At the second visit, participating students completed the established core questionnaire as well as a class-specific questionnaire of 5 to 7 co-created questions on factors influencing PA and SB based on the discussion during the first visit. The activPAL devices and parent (completed by one legal guardian) and teacher questionnaires were collected by the researchers. In the week after the second visit, participating students received a visualization of their individual accelerometer results. Between the second and third visit, the accelerometer data and class-specific questionnaires were analyzed by the researchers. During the third and final visit, researchers presented the overall class results and provided insight into the data analysis. Students provided input regarding their interpretation of the data, their thoughts on the extent to which the data are generalizable, and suggested next research steps. Finally, students and teachers had the opportunity to provide feedback on the experience and suggestions for improving future Citizen Science projects.

Since the field phase started in February 2020, it was affected by the COVID-19 pandemic, which was officially declared by the WHO on 11th of March 2020. Schools in Berlin and Brandenburg were closed for on-site teaching from March 22nd 2020 to May 3rd 2020 (first lockdown) and again (second lockdown) from December 16th 2020 to January 31st 2021. During the pandemic, remote learning was used as a measure to ensure continued schooling. Remote learning included individual learning from home with textbooks or learning materials complied by the teacher and also lessons conducted via virtual conferences. Two classes participated in this study before the pandemic, in February and March 2020; seven classes participated in September and October 2020 in full attendance; one class participated in January 2021 during the second lockdown while the schools were closed (visits were conducted virtually, and all devices and documents sent via mail). Two classes participated in May and June 2021, when schools were in the partial attendance mode, meaning that usually half of the class was present and the other half in home schooling.

**Fig. 2:**
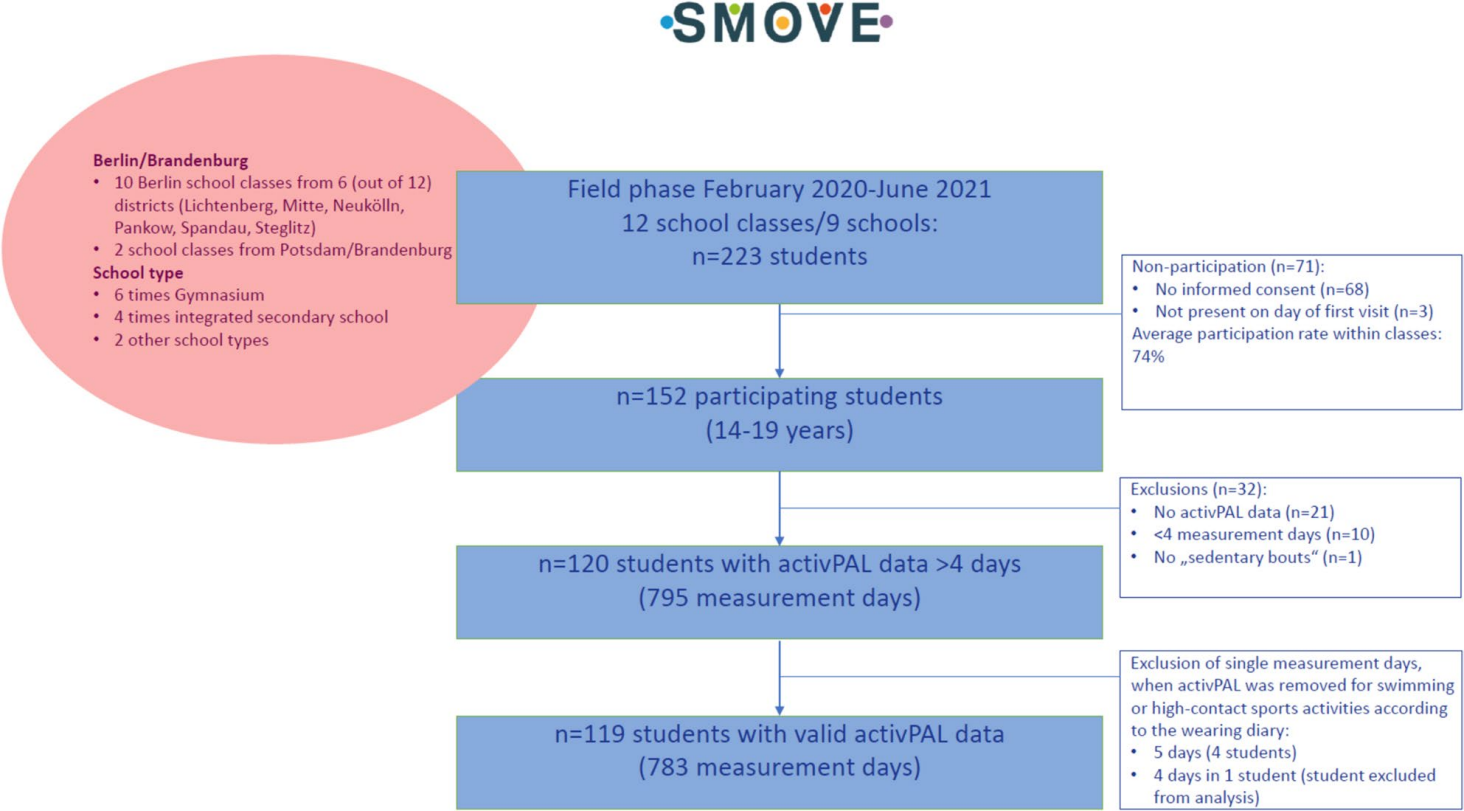
Flowchart of SMOVE study

Overall, twelve school classes took part in SMOVE and comprised overall 223 students of whom 152 participated in the data collection (Fig. 2). 

### Processing of accelerometry data

Activity data collected with the activPAL device was processed using proprietary algorithms (PALanalysis VANE algorithm, v8.11.8.75, PAL Technologies Ltd, Glasgow, Scotland) and a customized MatLab (version 7.0.1, Mathworks Inc, Natick, MA, USA) program which enabled the categorization of the amount of time spent sitting in bouts of varying length [21, 22]. For activity behaviors, in brief, the sum of the vector magnitude was extracted from triaxial accelerations measured at 20 Hz over 15 s epochs. A validated threshold for the sum of the vector magnitude was applied to quantify time spent in MVPA [22]. Epochs spent sitting, standing, and stepping were summed to provide time per 24 h spent in the respective activity type. Continuous null-counts >60 min during waking periods were considered as non-wear time. Waking periods were determined as the time between the rise time and bed time of participants. Rise time was identified by the first non-sedentary epoch after 5 a.m. and bed time was identified as the last non-sedentary epoch of the day followed by a prolonged sedentary period (>2 h). The primary activity-related variables of interest in the present analysis are time spent in MVPA (expressed in hours/day) as well as time spent sedentary (sitting or lying) during waking hours (hours/day).

### Qualitative analysis

In order to analyze and categorize the potential influencing factors of PA and SB that were collected by the 152 students during the group discussions in all twelve classes, we used inductive category forming [23]. After categorizing all potential influencing factors, a frequency analysis was performed to interpret the categories. The frequencies reflect, how many coded units (i.e., factors named by students) were assigned to the categories of the category scheme. It was assumed that the frequency with which each category appears is an indicator of the importance of that category with regard to the research question [23].

### Statistical analysis

For descriptive analyses, we calculated means and standard deviations (age) or absolute and relative frequencies for categorical variables overall and by sex. Accelerometer data was displayed as median and 25th and 75th percentile. For the co-created questions with numeric answers, minimum, maximum as well as median and 25th and 75th percentiles were extracted.

To investigate the association between personal, home-related, and school-related variables in relation to time spent in MVPA (minutes/day) and time spent sitting or lying during waking hours (sedentary waking minutes per day), multi-level linear regression analysis with random intercepts were used as described previously [14]. This method was chosen to control for dependence between measurement days within students. The first level consists of the daily accelerometer measurements, the second level consists of the individual participant. We analyzed potential influencing factors first in univariable models, followed by an analysis in models adjusted for age, sex and SES defined based on the parent’s education (ISCED 5, i.e. university degree or higher in either parent). For the analysis of potential influencing factors from the class-specific questionnaires, the underlying study populations for the questions differed, depending on how many classes each question was asked in. For the analysis of class-specific questions, univariable multilevel models were run, followed by multivariable models adjusted by age, sex, SES and school type. Given the limited sample size, we did not conduct sex-stratified analyses or test for interaction by sex. All analyses were conducted with SAS Enterprise Guide (version 8.3.2, SAS Institute, Inc., Cary, North Carolina) and two-sided p-values of 0.05 were set as level of significance.

## Results

The analysis dataset comprised 119 students providing *n* = 783 measurement days (Fig. 2). Of these, 91 (76.5%), 14 (11.8%), 6 (5.0%) and 8 (6.7%) students had 7, 6, 5 and 4 measurement days, respectively. For the analysis of the co-created questions, a further 7 participants were excluded due to missing class-specific questionnaires.

Characteristics of the study population are displayed in Table 1. At the time of the survey, the adolescents were on average 16 years old (range 14–19 years) with almost two-thirds self-identifying as female. and almost half from families with a high SES. More than half of students attended a Gymnasium (higher degree secondary school) and the vast majority attended schools in neighborhoods with teacher-reported high traffic safety. Median sedentary waking time per day was 10.8 h. Median time spent in MVPA was 40 min per day.

**Table 1:** Characteristics of the study population

	Total^a^	Female	Male
	[*n* = 119]	[*n* = 76, 63.9%]	[*n* = 42, 35.3%]
Age, years [mean (SD)]	16.0 (1.5)	16.2 (1.6)	15.8 (1.5)
Personal and home-related variables			
a.) Reported by parents			
Single parent household, [n(%)]	22 (18.5)	13 (17.1)	9 (21.4)
High socio-economic background^b^, [n(%)]	55 (46.2)	37 (48.7)	17 (40.5)
Immigrant background^c^, [n(%)]	28 (23.5)	17 (22.4)	10 (23.8)
b.) Reported by students			
Motivated by friends or family to be active, [n(%)]	37 (31.1)	27 (35.5)	10 (23.8)
Neighborhood encourages adolescents to be active outdoors, [n(%)]	65 (54.6)	36 (47.4)	28 (66.7)
Own smart phone, [n(%)]	118 (99.2)	76 (100.0)	41 (97.6)
Access to own computer/tablet, [n(%)]	108 (90.8)	70 (92.1)	37 (88.1)
Access to TV in own bedroom, [n(%)]	51 (42.9)	32 (42.1)	19 (45.2)
Rules for screen time in place, [n(%)]	29 (24.4)	15 (19.6)	13 (31.0)
School-related characteristics (reported by teachers)			
School type Gymnasium (preparatory school), [n(%)]	69 (58.0)	41 (53.9)	27 (64.3)
High traffic safety around school, [n(%)]	101 (84.9)	65 (85.5)	35 (83.3)
Access to sports equipment and/or dedicated areas for physical activities during break, [n(%)]	107 (89.9)	68 (89.5)	38 (90.5)
Students are allowed to use media devices during breaks, [n(%)]	47 (39.5)	29 (38.2)	18 (42.9)
Hours per day spent at school or on remote learning, [mean (SD)]	5.7 (1.0)	5.7 (1.1)	5.8 (0.9)
Triaxial activPAL data (on average per day)			
Steps, [median (25th; 75th percentile)]	7825 (6832;10587)	7834 (6777; 10168)	7744 (6832; 10966)
Sleep Hours, [median (25th; 75th percentile)]	8.6 (8.1; 9.3)	8.8 (8.1; 9.3)	8.4 (8.1; 9.1)
Sedentary Waking Hours, [median (25th; 75th percentile)]	10.8 (9.9; 11.5)	10.6 (9.8; 11.4)	11.0 (10.4; 11.9)
Standing Hours, [median (25th; 75th percentile)]	2.8 (2.4; 3.5)	3.0 (2.6; 3.7)	2.5 (2.0; 2.9)
Stepping Hours, [median (25th; 75th percentile)]	1.5 (1.3; 2.0)	1.5 (1.3; 1.8)	1.5 (1.3; 2.1)
MVPA^d^ Minutes, [median (25th; 75th percentile)]	40 (20; 55)	37 (28; 51)	41 (33; 63)
LIPA^e^ Minutes, [median (25th; 75th percentile)]	52 (41; 59)	52 (41; 59)	52 (40; 60)

Sleep Hours, Sedentary Waking Hours, Standing Hours, and Stepping Hours add up to 24 h. MVPA and LIPA add up to Stepping hours

^a^One student self-identified as non-binary and was excluded from sex-stratified analyses

^b^High SES is defined as ISCED (International Standard Classification of Education) ≥ 5

^c^Migrant background is defined as having at least one parent born outside of Germany

^d^Moderate-to-vigorous physical activity;

^e^Light intensity physical activity

The association between personal, home-related, and school-related variables and time spent in MVPA and sedentary waking time is displayed in Table 2. In univariable models, high versus low parental SES was associated with more time spent in MVPA (+ 11.2 min/day, 95%CI + 3.3, + 19.1), while age and sex were non-significantly associated with time spent in MVPA. In models adjusted for age, sex and parental SES, high traffic safety around school (+ 14.8 min/day; 95%CI + 0.9, + 28.7) and school type Gymnasium (versus other, + 10.7 min/day, 95% CI + 1.7, + 19.8) were positively associated with time spent in MVPA. Students who had access to their own TV in their bedroom spent less time in MVPA compared to those who had not (-7.7 min/day, 95% CI -14.9, -0.6), but this association was attenuated after adjustment for age, sex and parental SES. Access to own PC/tablet was non-significantly associated with less time spent in MVPA, while parental rules for screen time were not associated with time spent in MVPA or sedentary time. Among the personal and home-related factors, single-parent household, migration background, motivation to participate in physical activity by friends or family or an outdoor activity-friendly neighborhood were not associated with time spent in MVPA. Among the school-related factors, access to play equipment during breaks and permission to use media devices during breaks were not related to time spent in MVPA.

**Table 2:** Results from the multilevel models on the association between personal, home-related and school-related factors and daily time spent in moderate-to-vigorous physical activity and sedentary waking hours

		Time spent in moderate-to-vigorous physical activity in minutes per day		Sedentary waking time in minutes per day	
		Univariable	Age-sex-SES-adjusted	Univariable	Age-sex-SES-adjusted
Parameter	Comparison	β (95%CI)	β (95%CI)	β (95%CI)	β (95%CI)
Sex	female vs. male	-5.8 (-13.2; 1.5)		**-36.9 (-62.1; -11.8)**	
Age	per year	-2.1 (-4.4; 0.2)		0.2 (-7.8; 8.3)	
Socio-economic background (SES)	high vs. low	**11.2 (3.3; 19.1)**		-0.5 (-29.3; 28.3)	
Single-parent household	yes vs. no	-4.6 (-13.9; 4.7)	-1.6 (-11.0; 7.7)	6.5 (-26.1; 39.1)	3.7 (-29.7; 37.2)
Immigrant background of student	yes vs. no	2.5 (-6.2; 11.1)	2.5 (-6.9; 12.0)	7.8 (-22.4; 37.9)	13.0 (-20.6; 46.6)
Motivated by friends or family to be active	yes vs. no	-5.6 (-13.2; 2.0)	-5.5 (-12.9; 2.0)	2.1 (-24.7; 28.9)	8.0 (-18.6; 34.7)
Neighborhood encourages adolescents to be active outdoor	disagree vs. agree	-10.9 (-33.5; 11.6)	-3.7 (-25.8; 18.4)	-29.1 (-107.9; 49.7)	-21.2 (-101; 58.2)
	neutral vs. agree	1.9 (-5.3; 9.2)	4.0 (-3.2; 11.3)	-0.9 (-26.3; 24.5)	7.7 (-18.3; 33.7)
Access to own PC/tablet	yes vs. no	-10.8 (-23.6; 2.0)	-10.6 (-23.5; 2.2)	22.8 (-20.3; 65.9)	19.2 (-25.1; 63.5)
Access to TV in own bedroom	yes vs. no	**-7.7 (-14.9; -0.6)**	-5.6 (-12.9; 1.6)	-0.1 (-25.6; 25.4)	2.7 (-23.6; 28.9)
Rules for screen time in place	yes vs. no	1.7 (-6.6; 10.0)	-2.4 (-11.2; 6.4)	-3.7 (-31.8; 24.5)	-5.5 (-35.8; 24.7)
Traffic safety around school	high vs. low	**15.3 (2.8; 27.8)**	**14.8 (0.9; 28.7)**	-1.1 (-45.8; 43.6)	-8.9 (-59.5; 41.7)
Students have access to play equipment during breaks	agree vs. disagree	-0.5 (-20.7; 19.7)	-2.8 (-23.4; 17.8)	14.1 (-56.7; 85.0)	16.1 (-57.9; 90.1)
Students are allowed to use media devices during breaks	yes vs. no	-5.6 (-12.9; 1.8)	-5.2 (-13.0; 2.6)	-6.4 (-32.4; 19.5)	-9.2 (-37.2; 18.8)
School type	Gymnasium (preparatory school) vs. other school types	**12.6 (5.8; 19.5)**	**10.7 (1.7; 19.8)**	-8.5 (-33.6; 16.5)	-19.3 (-52.3; 13.6)

Figures in bold refer to statistically significant findings (*p* < 0.05)

When investigating time spent sedentary, we observed that girls spent less time sedentary (-36.9 min/day, 95% CI -62.1, -11.8) compared to boys. All other person-, home- and school-related factors investigated were not associated with sedentary time.

**Fig. 3:**
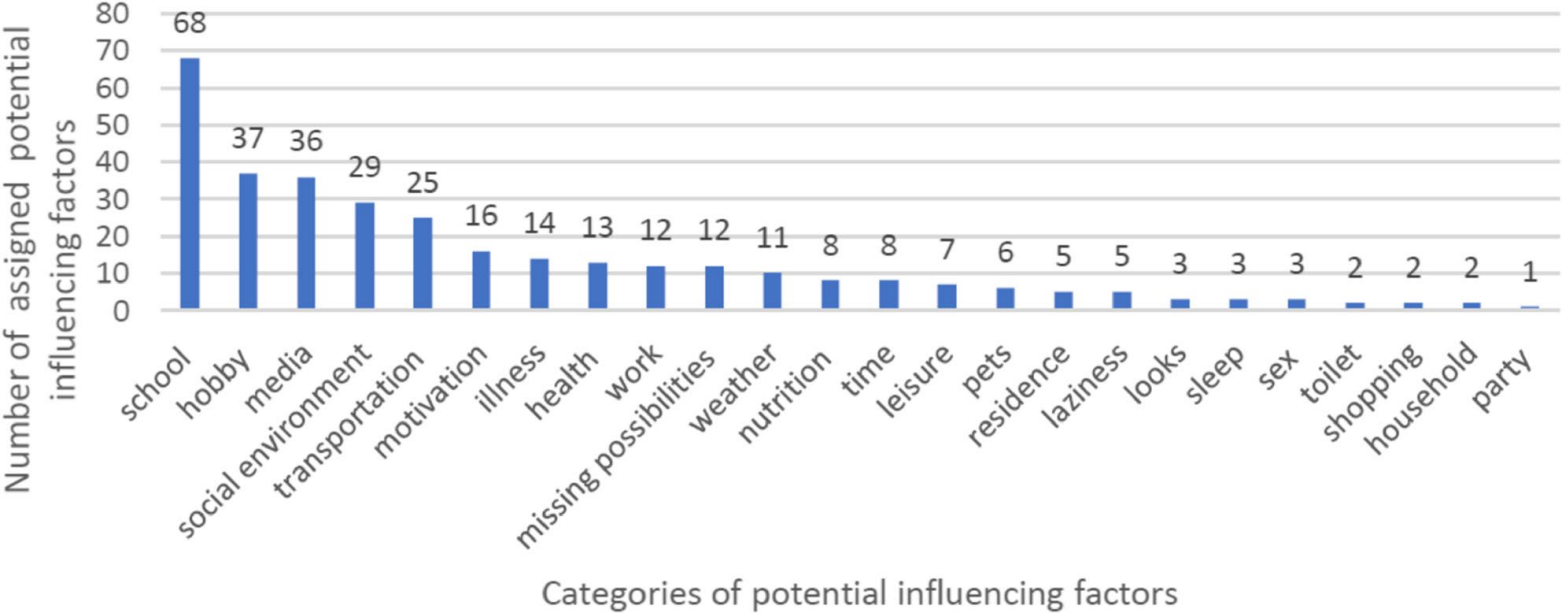
Categories of potential influencing factors named by students participating in SMOVE, sorted according to how often the factors were named

The qualitative analysis of the factors named by students as potential influencing factors of their PA or SB is shown in Fig. 3. The total number of potential influencing factors collected by the students was 328. Based on these factors, 24 categories were created. The frequency analysis demonstrated that the most frequently named factors were school-related (21%), followed by factors related to students’ hobbies (12%), their (social) media use (11%), their social environment (9%), and their way of transportation (8%).

Table 3 gives an overview of all co-created questions from the class-specific questionnaires as well as the number of classes/students who answered these questions. The distributions of the students’ responses are shown in Supplemental Table 1 for categorical variables and in Supplemental Table 2 for continuous variables.

**Table 3:** Co-created questions on potential influencing factors of physical activity and sedentary behavior and number of classes and students each question was asked in

Question	Category	*N* (classes)	*n* (students)
How much time do you spend on homework? Separate questions for school day and weekend day	school	8	97
I´m bothered by the long hours of sitting in school	school	6	74
After school and doing my homework I feel exhausted	school	5	56
On an average school day or weekend day, how much time do you spend outside of class studying? Separate questions for school day and weekend day	school	2	25
On an average school day or weekend day, how much time do you spend on social media? Separate questions for school day and weekend day	media	7	90
I feel physically fit	health	6	70
When I have a choice in everyday life, I opt for the more physically active behavior	health	1	6
The physical activity and sports behavior of my friends influences my own	social environment	5	52
I feel like I don´t have enough time	time	4	46
I am motivated to be physically active	motivation	4	37
I have the possibility (spatially, financially) to do the type of sport I want to do	missing possibilities	3	31
How much time did you spend on sports on average per week in the past 12 months? Separate questions for summer and winter	weather	2	26
My hobbies make me more physical active	hobby	2	20
I am more physically active during vacation than during school times	hobby	1	20
I regularly work out at the gym	hobby	1	15
Do you have a pet? Separate questions on type of pet	pets	2	19
I´m satisfied with my body	looks	1	15

In the multivariable analysis of class-specific questions, student’s own motivation was identified as an influencing factor for time spent in MVPA in the univariable model: Compared to students who agreed to being motivated to be physically active, those who strongly agreed spent more time in MVPA (+ 22.5 min/day; 95% CI 1.7, 43.3) and those who neither agreed nor disagreed spent less time in MVPA (-16.3 min/day; 95% CI -31.0, -1.6; Supplemental Table 3). These findings were statistically non-significant after multivariable adjustment for age, sex and parental SES. Students who strongly agreed to feeling exhausted after school and homework spent less time in MVPA (-15.3 min/day; 95% CI -30.3, -0.4) compared to those who agreed in the multivariable model. Compared to students who strongly agreed to regularly working out at the gym, those who strongly disagreed (-33.2; 95% CI -59.3, -7.2) or disagreed (-30.0; 95% CI -60.1, 0.0) spent significantly less time in MVPA in the multivariable adjusted model. Time spent with homework or social media on either weekend or school days were not associated with time spent in MVPA, but time spent outside of class studying on weekends was associated with more time spent in MVPA in the multivariable model. In the univariable models, body satisfaction, feeling of physical fitness and hobbies that make more physically active showed some associations with time spent in MVPA. None of the class-specific questions showed associations with time spent sedentary. Overall, sample sizes were limited for the analyses of class-specific questions in relation to time spent in MVPA or time spent sedentary.

## Discussion

In this school-based Citizen Science study, we investigated influencing factors of device-based measured PA and SB based on established potential influencing factors as well as with co-created class-specific questionnaires. We observed that higher parental SES, higher degree school type and perceived high traffic safety around school was associated with more time spent in MVPA, while access to media devices was associated with less time spent in MVPA. Among the newly developed questionnaire items, student’s own motivation to be physically active, active hobbies and reported regular gym visits were associated with more time spent in MVPA, while feeling exhausted after school and homework was associated with less time spent in MVPA. Time spent sedentary was not influenced by most factors investigated here, with the exception of female sex, which was associated with less time spent sedentary.

The findings presented here suggest that both parental SES and attending a Gymnasium (i.e. higher degree school type), which is related to parental educational level in Germany [24], were associated with more time spent in MVPA but not with time spent sedentary. Previous studies found mixed evidence for an association between parental SES and PA in adolescents [25, 26]. In a large school-based survey among more than 10,000 students from six European cities (including Hannover, Germany), higher SES was associated with more questionnaire-based vigorous PA [27]. In contrast, in the European HELENA study, with more than 2,000 adolescents and device-based measurement of PA and SB (hip-worn ActiGraph), no differences in PA or SB by maternal education was observed [28]. Thus, more research into the association of parental SES with device-based measures of PA and SB is warranted, specifically addressing whether there are country-specific differences.

Our finding that higher traffic safety around school (as measured by teachers’ perception) is associated with more time spent in MVPA seems plausible as a safe environment could encourage active transport to school and outdoor activities in general. In line with our findings, traffic safety around school has been positively related to prospective one-year increases in accelerometer-measured moderate as well as vigorous PA in a UK study of children aged 10–11 years [29]. Home neighborhood traffic safety has been positively related to PA in adolescents in previous (largely questionnaire-based) research [13], although in our study, a home neighborhood encouraging adolescents to be active outdoors (reported by students) was not associated with MVPA. In contrast to previous evidence [13], access to play equipment during school breaks was not related to time spent in MVPA in our study. These inconsistent findings across literature warrant clarification of the role of neighborhood characteristics as influencing factors of PA and SB in larger samples employing device-based measures of PA and SB. Furthermore, the incorporation of either/or time-based or geolocation-based Ecological Momentary Assessment (via smartphone technology) with device-based PA and SB measurement may also add further insights as to what influences PA and SB in specific settings (i.e., while playing in neighborhood) and at specific times (i.e., during school breaks) [30]. The here identified factors were associated with differences in daily MVPA between 10 (school type) and 15 min per day (traffic safety around school), which is within the range of physiological importance. For example, it has been shown that 27 min per day more time spent in MVPA at age 15 years is associated with improved cardiometabolic risk factors such as maximal oxygen uptake, VO_2max_, fasting insulin and visceral fat at age 24 years [31].

For participants in this study, having a TV in their own bedroom, as well as access to their own PC or tablet, were associated with less time spent in MVPA. In contrast, parental rules regarding screen time were not related to time spent in PA. While access to own TV or or PC/tablet are only indicators for daily screen time, our findings are supported by studies that related screen time to PA measures and also observed inverse associations [32, 33]. Interestingly, screen-related factors were not associated with sedentary time in our study. The findings reported here and in the literature suggests that more research is needed on gaining an understanding of the influencing factors of sedentary (screen-based and other) time [34].

Time spent sedentary during waking hours was not related to the factors investigated in this study, but lower sedentary hours in girls versus boys were observed. Although differences were small, this finding is in contrast to the German GINIplus [35] and the European HELENA study [28], where higher sedentary times in girls than in boys were apparent. The discrepant findings may be related to the fact that in the reported studies, accelerometers were worn only during waking hours and removed at night. In addition, both studies used hip-worn accelerometers compared to the thigh-worn activPAL that was used in the present study. The activPAL has previously been identified as a highly accurate device for measuring SB, in particular due to its ability to distinguish between sitting and standing time [20, 36]. Further research using high-quality measures of sedentary time, which also focuses on how sedentary time is accumulated (i.e., sedentary bout durations in different durations and settings) should be conducted to better understand the behavior and its influencers.

The qualitative analysis of factors named by students as potential influencing factors of their PA and SB shows that students perceive school, hobbies and social media as having a substantial impact, which is in line with previous qualitative research [37–39]. Based on our Citizen Science approach these factors pose key domains that should be considered in future public health research. In our study, sample size was limited to investigate the association between the co-created novel questions on potential influencing factors and their association with PA and SB. We identified motivation, feeling exhausted after school and homework, body satisfaction, physically active hobbies and especially reporting of regular gym visits as potential influencing factors of PA, although not all associations were statistically significant after multivariable adjustment for age, sex and parental SES. Internal motivation to be physically active was associated with more time spent in MVPA in the class-specific questionnaire. In the established questionnaire, regular external motivation by friends or family to do sports was reported by one third of the students, but this variable was not related to time spent in MVPA. Thus, based on our findings, internal motivation may be a key influence factor of PA, while external motivation may play a minor role for actual time spent in MVPA. These observations are in line with previous studies showing that intrinsic motivation is associated with higher PA levels [40, 41], while controlled forms of motivation (e.g. acting to get a reward or avoid punishment, or acting because of feelings of guilt) showed weak or inverse associations with PA in adolescents [42].

Reported body satisfaction was positively associated with PA in our study, which is in line with a systematic review considering the evidence from 28 studies conducted in adolescents [43]. In addition, the observations that students who reported that their hobbies make them more physically active and/or reported regular gym visits spent more time in MVPA are in line with studies among students that showed that students participating in physically active hobbies, such as organized sports participation, spent more time in MVPA [44, 45]. Other co-created questions assessing school-related, hobby-related and social media-related and personal influencing factors, were not associated with time spent in MVPA or sedentary time in our study. However, because the co-created questions were class-specific, the sample size was limited and investigation of these potential influencing factors in larger samples is needed.

Strengths of the present study include the 24 h per day device-based measurement of PA over one full week with a thigh-worn accelerometer that is specifically strong in distinguishing standing and sitting/lying behavior. Furthermore, the use of both established and newly developed questions on potential influencing factors is a strength of this study. Nevertheless, there are limitations: the cross-sectional analysis precludes a causal interpretation of observed associations. In addition, the sample size was limited and investigation, particularly of the newly developed co-created questions, in larger sample sizes in needed. Since the field phase of the study coincided with the COVID-19 pandemic, a smaller sample size than planned was achieved and we cannot exclude that observed associations between influencing factors and PA and SB could have been affected by the pandemic. With almost two-thirds, girls were overrepresented in our study population. We adjusted our analyses by sex but cannot exclude that this sex-imbalance affects our findings and it also limits the generalizability of our findings. Furthermore, while we were able to adjust our analyses for major confounders such as age, sex and SES, we cannot exclude residual confounding including by potential confounders that were not assessed (e.g. information on siblings). In addition, this study was conducted among students from Berlin and Brandenburg and may not be generalized to other populations.

In conclusion, this school-based study showed that parental SES, school type, access to media devices as well as internal motivation to be physically active and the feeling of exhaustion after school and homework are influencing factors of time spent in MVPA, while sedentary behavior was not influenced by factors investigated in this study. Our findings, which warrant confirmation in larger and more representative samples, point to potential action points that may be targeted in public health interventions aimed at increasing PA to improve health in adolescents. The questionnaire-items developed by students and scientist in a co-creative Citizen Science approach enrich the investigation of potential influencing factors of PA and SB. Future research with a more detailed assessment of internal motivation, feeling exhausted after school and homework as well body satisfaction and larger sample sizes is warranted in order to develop targeted intervention strategies aiming at increasing physical activity levels in adolescents. Furthermore, the identification of SES and school type as determinants of PA encourages the focus on vulnerable groups prone to low PA for interventions aiming at increasing PA. Finally, our study demonstrated that it is feasible to involve students directly in the questionnaire-development of epidemiological studies.

## Supplementary Information

Supplementary Material 1.

## Data Availability

Access to the data can be granted upon request to the corresponding author.

## Abbreviations

MVPA: Moderate-to-vigorous physical activity
PA: Physical activity
SB: Sedentary behavior
SES: Socioeconomic status
SMOVE: Science that makes me move
WHO: World Health Organization
