# Supplementary material for: Factors associated with device-based measured physical activity and sedentary behavior in a cross-sectional citizen science study of adolescents during the COVID-19 pandemic
Source: BMC Public Health. 2025 Dec 5;26:58. doi: 10.1186/s12889-025-25776-5 (PMC12771833; doi:10.1186/s12889-025-25776-5)
Supplement: Supplementary file 1 — Supplementary Material 1. [file 12889_2025_25776_MOESM1_ESM.docx]

**Factors associated with device-based measured physical activity and sedentary behavior in a cross-sectional Citizen Science study of adolescents**

**Authors: Katharina Nimptsch, Yang Jiao, Lethizia Oliver-Stanley, Jonas Widmann, Lina Jaeschke, Astrid Steinbrecher, Kieran Dowd, Tobias Pischon**

**Corresponding author:**

Dr. Katharina Nimptsch

Molecular Epidemiology Research Group

Max Delbrück Center for Molecular Medicine (MDC)

Robert-Rössle-Straße 10

13125 Berlin

Tel.: ++49 30 / 9406 – 4573

Fax: ++49 30 / 9406 – 4576

e-mail: katharina.nimptsch@mdc-berlin.de

**Supplementary file: Supplemental tables 1–3**

**Supplemental table 1.** Categorical questions included in the class-specific questionnaires and the distribution of student’s responses for each question

| **Question** | **N (class)** | **N (students)** |  |  |  |  |  |  |
| --- | --- | --- | --- | --- | --- | --- | --- | --- |
|  |  |  |  | **Disagree very strongly** | **Disagree** | **Neither agree nor disagree** | **Agree** | **Agree very strongly** |
| **I´m more physical active during my spare time than in school** | 1 | 20 | Frequency | **3** | **5** | **3** | **4** | **5** |
|  |  |  | Percent | 15% | 25% | 15% | 20% | 25% |
| **I´m satisfied with my body** | 1 | 15 | Frequency | **0** | **3** | **5** | **3** | **4** |
|  |  |  | Percent | 0% | 20% | 33.33% | 20% | 26.67% |
| **I´m bothered by the long hours of sitting in school** | 6 | 74 | Frequency | **1** | **6** | **21** | **34** | **12** |
|  |  |  | Percent | 1.35% | 8.11% | 28.38% | 45.95% | 16.22% |
| **After school and doing my homework I feel exhausted** | 5 | 56 | Frequency | **0** | **3** | **7** | **35** | **11** |
|  |  |  | Percent | 0% | 5.36% | 12.50% | 62.50% | 19.64% |
| **I feel physically fit** | 6 | 70 | Frequency | **0** | **11** | **22** | **22** | **15** |
|  |  |  | Percent | 0% | 15.71% | 31.43% | 31.43% | 21.43% |
| **I feel like I don´t have enough time** | 4 | 46 | Frequency | **0** | **7** | **13** | **20** | **6** |
|  |  |  | Percent | 0% | 15.22% | 28.26% | 43.48% | 13.04% |
| **The physical activity and sports behavior of my friends influences my own** | 5 | 52 | Frequency | **6** | **14** | **14** | **15** | **3** |
|  |  |  | Percent | 11.54% | 26.92% | 26.92% | 28.85% | 5.77% |
| **My hobbies make me more physical active** | 2 | 20 | Frequency | **1** | **5** | **0** | **3** | **11** |
|  |  |  | Percent | 5% | 25% | 0% | 15% | 55% |
| **I am motivated to be physically active** | 4 | 43 | Frequency | **1** | **5** | **15** | **16** | **6** |
|  |  |  | Percent | 2.33% | 11.63% | 34.88% | 37.21% | 13.95% |
| **I have the possibility (spatially, financially) to do the type of sport I want to do** | 3 | 31 | Frequency | **0** | **1** | **5** | **13** | **12** |
|  |  |  | Percent | 0% | 3.23% | 16.13% | 41.94% | 38.71% |
| **I regularly work out at the gym** | 1 | 15 | Frequency | **5** | **5** | **3** | **0** | **2** |
|  |  |  | Percent | 33.33% | 33.33% | 20.00% | 0% | 13.33% |
| **When I have a choice in everyday life, I opt for the more physically active behavior** | 1 | 6 | Frequency | **0** | **1** | **2** | **3** | **0** |
|  |  |  | Percent | 0% | 16.67% | 33.33% | 50% | 0% |
|  |  |  |  | **Yes** | **No** | **Does not apply** |  |  |
| **Do you have a pet?** | 2 | 19 | Frequency | **14** | **5** |  |  |  |
|  |  |  | Percent | 73.68% | 26.32% |  |  |  |
| **If you have a pet, in which category does it fit best?- Cat** | 2 | 19 | Frequency | **6** | **8** | **5** |  |  |
|  |  |  | Percent | 31.58% | 42.11% | 26.32% |  |  |
| **If you have a pet, in which category does it fit best?- Dog** | 2 | 19 | Frequency | **7** | **7** | **5** |  |  |
|  |  |  | Percent | 36.84% | 36.84% | 26.32% |  |  |
| **If you have a pet, in which category does it fit best?- Horse** | 2 | 19 | Frequency | **0** | **14** | **5** |  |  |
|  |  |  | Percent | 0 | 73.68% | 26.32% |  |  |
| **If you have a pet, in which category does it fit best?- Small animal (rodent, reptile, fish, bird...)** | 2 | 19 | Frequency | **4** | **10** | **5** |  |  |
|  |  |  | Percent | 21.05% | 52.63% | 26.32% |  |  |
| **If you have a pet, in which category does it fit best?- Other** | 2 | 19 | Frequency |  |  | **19** |  |  |
|  |  |  | Percent |  |  | 100% |  |  |

**Supplemental table 2.** Continuous questions included in the class-specific questionnaires and the distribution of responses for each question

| **Question** | **N (class)** | **N (students)** |  | **N** | **Minimum** | **Maximum** | **Median** | **25th Pctl** | **75th Pctl** | **Missing** |
| --- | --- | --- | --- | --- | --- | --- | --- | --- | --- | --- |
| **How much time do you spend on homework on an average school day?** | 7 | 87 |  | **all (86)** | **0** | **480** | **60** | **30** | **90** | 1 |
|  |  |  | minutes/day | female (54) | 5 | 480 | 60 | 45 | 90 |  |
|  |  |  |  | male (31) | 0 | 240 | 30 | 20 | 60 |  |
| **How much time do you spend on homework on an average weekend day?** | 8 | 97 |  | **all (95)** | **0** | **780** | **60** | **20** | **105** | 2 |
|  |  |  | minutes/day | female (60) | 0 | 610 | 60 | 30 | 120 |  |
|  |  |  |  | male (35) | 0 | 780 | 30 | 15 | 60 |  |
| **On an average school day or weekend day, how much time do you spend outside of class studying? - School day** | 2 | 25 |  | **all (25)** | **0** | **440** | **60** | **20** | **120** |  |
|  |  |  | minutes/day | female (13) | 20 | 240 | 120 | 60 | 150 |  |
|  |  |  |  | male (12) | 0 | 120 | 25 | 15 | 60 |  |
| **On an average school day or weekend day, how much time do you spend outside of class studying? - Weekend day** | 2 | 25 |  | **all (25)** | **0** | **300** | **95** | **40** | **180** |  |
|  |  |  | minutes/day | female (13) | 30 | 300 | 120 | 60 | 180 |  |
|  |  |  |  | male (12) | 0 | 240 | 42.5 | 5 | 140 |  |
| **On an average school day or weekend day, how much time do you spend on social media? - School day** | 4 | 62 |  | **all (62)** | **10** | **360** | **120** | **60** | **180** |  |
|  |  |  | minutes/day | female (36) | 20 | 360 | 120 | 60 | 180 |  |
|  |  |  |  | male (26) | 10 | 360 | 110 | 50 | 180 |  |
| **On an average school day or weekend day, how much time do you spend on social media? - School day (classroom teaching)** | 3 | 28 |  | **all (24)** | **10** | **830** | **165** | **40** | **300** | 4 |
|  |  |  | minutes/day | female (12) | 20 | 480 | 165 | 67.5 | 240 |  |
|  |  |  |  | male (12) | 10 | 830 | 160 | 25 | 410 |  |
| **On an average school day or weekend day, how much time do you spend on social media? - School day (homeschooling)** | 3 | 28 |  | **all (25)** | **0** | **1100** | **230** | **40** | **300** | 3 |
|  |  |  | minutes/day | female (13) | 0 | 420 | 240 | 90 | 300 |  |
|  |  |  |  | male (12) | 10 | 1100 | 175 | 18 | 375 |  |
| **On an average school day or weekend day, how much time do you spend on social media? - Weekend** | 7 | 91 |  | **all 88)** | **10** | **1000** | **180** | **70** | **300** | 3 |
|  |  |  | minutes/day | female (49) | 20 | 540 | 180 | 120 | 270 |  |
|  |  |  |  | male (38) | 10 | 1000 | 180 | 45 | 300 |  |
| **How much time did you spend on sports on average per week in the past 12 months? - In summer** | 2 | 26 |  | **all (26)** | **80** | **1680** | **440** | **300** | **650** |  |
|  |  |  | minutes/week | female (13) | 80 | 1680 | 360 | 300 | 650 |  |
|  |  |  |  | male (13) | 200 | 800 | 480 | 300 | 610 |  |
| **How much time did you spend on sports on average per week in the past 12 months? - In winter** | 2 | 26 |  | **all (26)** | **0** | **1680** | **305** | **180** | **600** |  |
|  |  |  | minutes/week | female (13) | 100 | 1680 | 300 | 180 | 600 |  |
|  |  |  |  | male (13) | 0 | 680 | 360 | 240 | 540 |  |

Numbers of males and females do not add up to the numbers of all if the student who self-identified as non-binary was included in this question.

**Supplemental table 3.** Results of multilevel regression analysis between co-created potential influencing factors and and daily time spent in moderate-to-vigorous physical activity and sedentary waking hours

|  |  |  | **Time spent in moderate-to-vigorous physical activity in minutes per day** | |  | **Sedentary waking time in minutes per day** | |
| --- | --- | --- | --- | --- | --- | --- | --- |
|  |  |  | **Univariable** | **Age-sex-SES-adjusted** |  | **Univariable** | **Age-sex-SES-adjusted** |
| **Potential influencing factors** | |  | **β (95%CI)** | **β (95%CI)** |  | **β (95%CI)** | **β (95%CI)** |
|  | **Comparison** | **N^1^** |  |  |  |  |  |
| **I´m more physical active during my spare time than in school** | | 18 |  |  |  |  |  |
|  | Disagree vs. agree |  | **-22.6 (-42.7; -2.5)** | **-22.7 (-42.2; -3.1)** |  | -4.4 (-71.9; 63.1) | 35.5 (-41.4; 112.3) |
|  | Neutral vs. agree |  | -20.7 (-47.0; 5.5) | -59.1 (-98.3; -19.9) |  | 36.3 (-51.9; 124.4) | 73.5 (-80.6; 227.6) |
| **I´m satisfied with my body** | | 13 |  |  |  |  |  |
|  | Disagree vs. agree |  | **-31.5 (-53.1; -9.8)** | -14.3 (-46.3; 17.6) |  | -0.2 (-86.7; 86.3 | 42.3 (-67.5; 152.2) |
|  | Neutral vs. agree |  | **-20.9 (-37.4; -4.4)** | -5.6 (-31.3; 20.1) |  | -32.5 (-97.7; 32.8) | -11.4 (-99.9; 77.0) |
| **I´m bothered by the long hours of sitting in school** | | 64 |  |  |  |  |  |
|  | Disagree vs. agree |  | -3.1 (-20.4; 14.1) | -7.9 (-24.7; 9.0) |  | 57.7 (0.9; 114.6) | 49.8 (-8.5; 108.2) |
|  | Neutral vs. agree |  | -0.6 (-12.2; 11.0) | -2.2 (-14.4; 10.0) |  | 9.3 (-29.0; 47.5) | 8.8 (-33.5; 51.1) |
|  | Strongly agree vs. agree |  | 9.7 (-4.9; 24.4) | 8.2 (-7.5; 24.0) |  | 13.7 (-34.6; 62.0) | 41.2 (-13.2; 95.7) |
| **After school and doing my homework I feel exhausted** | | 43 |  |  |  |  |  |
|  | Disagree vs. agree |  | **-36.8 (-72.8; -0.7)** | -29.1 (-71.7; 13.4) |  | -10.3 (-54.4; 33.8) | -23.8 (-154; 106.4) |
|  | Neutral vs. agree |  | 16.4 (-3.2; 36.0) | 12.4 (-11.9; 36.7) |  | 17.5 (-46.0; 80.9) | -8.2 (-82.7; 66.4) |
|  | Strongly agree vs. agree |  | **-14.0 (-27.7; -0.3)** | **-15.3 (-30.3; -0.4)** |  | -10.3 (-54.4; 33.8) | 4.0 (-41.9; 49.8) |
| **I feel physically fit** | | 60 |  |  |  |  |  |
|  | Disagree vs. agree |  | **-18.7 (-33.3; -4.1)** | -13.8 (-29.8; 2.2) |  | 26.0 (-25.4; 77.4) | 23.0 (-29.9; 75.8) |
|  | Neutral vs. agree |  | -5.4 (-17.6; 6.8) | -3.8 (-17.2; 9.6) |  | 18.1 (-24.7; 61.0) | 18.0 (-26.3; 62.4) |
|  | Strongly agree vs. agree |  | 2.5 (-11.0; 15.9) | 2.5 (-11.8; 16.8) |  | 30.3 (-17.0; 77.7) | 24.1 (-23.1; 71.3) |
| **I feel like I don´t have enough time** | | 39 |  |  |  |  |  |
|  | Disagree vs. agree |  | -6.3 (-24.9; 12.2) | -12.1 (-31.0; 6.7) |  | 27.0 (-36.3; 90.3) | 45.3 (-26.9; 117.4) |
|  | Neutral vs. agree |  | -3.1 (-19.1; 13.0) | -3.1 (-18.4; 12.2) |  | -5.0 (-59.9; 49.8) | 19.1 (-39.6; 77.8) |
|  | Strongly agree vs. agree |  | -14.2 (-33.8; 5.4) | -17.1 (-35.0; 0.7) |  | -13.2 (-80.2; 53.9) | -12.3 (-80.5; 55.9) |
| **The physical activity and sports behavior of my friends influences my own** | | 43 |  |  |  |  |  |
|  | Strongly disagree vs. agree |  | -17.0 (-39.4; 5.3) | 1.0 (-24.6; 26.6) |  | 57.7 (-16.9; 132.3) | 35.0 (-58.1; 128.0) |
|  | Disagree vs. agree |  | -10.4 (-25.3; 4.6) | -5.6 (-23.0; 11.8) |  | 47.9 (-1.8; 97.7) | 35.6 (-27.8; 99.0) |
|  | Neutral vs. agree |  | -2.1 (-18.1; 14.0) | 3.0 (-14.2; 20.3) |  | 44.0 (-9.5; 97.5) | 27.0 (-35.7; 89.7) |
| **My hobbies make me more physical active** | | 13 |  |  |  |  |  |
|  | Disagree vs. agree |  | **-17.4 (-33.8; -1.0)** | -15.8 (-42.3; 10.8) |  | 54.6 (-3.7; 112.8) | 68.6 (-18.7; 155.8) |
| **I am motivated to be physically active** | | 31 |  |  |  |  |  |
|  | Disagree vs. agree |  | 1.4 (-17.3; 20.1) | 7.7 (-13.5; 28.9) |  | -35.0 (-100; 30.2) | -19.4 (-90.4; 51.6) |
|  | Neutral vs. agree |  | **-16.3 (-31.0; -1.6)** | -8.0 (-26.3; 10.3) |  | 3.7 (-47.4; 54.7) | 13.6 (-47.5; 74.8) |
|  | Strongly agree vs. agree |  | **22.5 (1.7; 43.3)** | 29.1 (-0.1; 58.4) |  | 19.1 (-53.7; 91.8) | -4.4 (-103; 94.0) |
| **I have the possibility to do the type of sport I want to do** | | 26 |  |  |  |  |  |
|  | Disagree vs. agree |  | -10.1 (-48.7; 28.6) | -28.0 (-83.5; 27.5) |  | -27.9 (-145; 89.6) | -2.7 (-163; 157.2) |
|  | Neutral vs. agree |  | -18.0 (-39.7; 3.7) | 1.9 (-26.2; 30.0) |  | -3.7 (-69.6; 62.2) | -14.7 (-95.8; 66.5) |
|  | Strongly agree vs. agree |  | 1.1 (-15.4; 17.5) | -4.5 (-23.8; 14.9) |  | -2.4 (-52.5; 47.8) | -29.6 (-85.4; 26.2) |
| **I regularly work out at the gym** | | 13 |  |  |  |  |  |
|  | Strongly disagree vs. strongly agree |  | **-37.5 (-59.0; -16.1)** | **-33.2 (-59.3; -7.2)** |  | 3.6 (-93.0; 100.2) | 38.2 (-47.4; 123.8) |
|  | Disagree vs. strongly agree |  | **-34.3 (-55.1; -13.5)** | **-30.0 (-60.1; 0.0)** |  | -1.7 (-95.2; 91.8) | 117.0 (18.4; 215.6) |
|  | Neutral vs. strongly agree |  | -16.2 (-41.3; 9.0) | -12.4 (-45.0; 20.2) |  | -45.3 (-157.9; 67.3) | 25.6 (-81.3; 132.6) |
| **Do you have a pet?** | | 17 |  |  |  |  |  |
|  | Yes vs. no |  | 2.1 (-16.9; 21.2) | 11.5 (-13.3; 36.3) |  | 19.6 (-45.5; 84.7) | 2.2 (-84.1; 88.4) |
| **Time spent on homework (school day) (h/day)** | | 76 | -2.8 (-6.0; 0.3) | -1.9 (-5.1; 1.3) |  | 6.2 (-2.7; 15.1) | 8.2 (-0.8; 17.2) |
| **Time spent on homework (weekend day) (h/day)** | | 69 | -1.2 (-3.1; 0.7) | -0.9 (-2.9; 1.1) |  | 2.1 (-4.6; 8.7) | 2.1 (-4.3; 8.6) |
| **Time spent outside of class studying (school day) (h/day)** | | 20 | 0.9 (-8.5; 10.2) | 2.3 (-9.3; 13.8) |  | **-23.0 (-41.4; -4.6)** | -8.9 (-39.4; 21.6) |
| **Time spent outside of class studying (weekend day) (h/day)** | | 20 | **7.1 (0.4; 13.9)** | **9.1 (2.2; 15.9)** |  | **-27.2 (-50.3; -4.1)** | -31.7 (-63.7; 0.4) |
| **Time spent on social media (school day) (h/day)** | | 56 | 1.6 (-2.5; 5.7) | 1.9 (-2.2; 6.0) |  | -8.9 (-19.5; 1.6) | -5.6 (-17.5; 6.4) |
| **Time spent on social media (weekend day) (h/day)** | | 74 | 0.1 (-1.3; 1.5) | -0.2 (-2.1; 1.7) |  | -3.8 (-9.2; 1.6) | -5.2 (-12.1; 1.7) |
| **Time spent on sports in the past 12 month (summer) (h/week)** | | 17 | -0.4 (-2.2; 1.4) | -1.5 (-4.4; 1.3) |  | -1.2 (-5.8; 3.4) | 3.8 (-2.2; 9.8) |
| **Time spent on sports in the past 12 month (winter) (h/week)** | | 17 | -0.3 (-2.1; 1.4) | -1.3 (-3.8; 1.2) |  | -0.5 (-5.0; 3.9) | 4.0 (-0.9; 8.9) |

^1^ Number of students included in analysis
